# The Medical Schools

**Published:** 1904-09-03

**Authors:** 


					The Medical Schools.
sK choice of a medical school is a matter that
ould receive a great deal of attention at the hands
the parents, guardians, or advisers of intending
radical students. Medical schools vary considerably
.Q their tone, in their traditions, in the quality of
Qstruction provided, and in many other ways, and
ultimate success in life of an intending medical
Practitioner may hang to a large extent on the choice
01 Ms school.
1 ^kile London will probably always occupy the
fading place in the United Kingdom as a centre of
abl e<^ucation, it does not follow that it is invari-
^Jy the best place for a prospective medical student,
-veral^ of the large provincial towns possess
^Ij^hhag medical schools and can claim as their
udren some of the leading men in the profession,
u> indeed, they possess many advantages which
are not found in the metropolis. The size of a
hospital is not of great importance for teaching
purposes; of more consequence is the size of the
medical school. Other things being equal, a large
school is usually better than a small one, because it
is better able to supply well equipped laboratories,
and first-rate teachers.
It may be said that it is impossible to generalise
much further than this on the question of the choice
of a school. Individual requirements are probably
the surest guides.
Under the head " composition fee " we have men-
tioned the sum required by each hospital and medical
school for all the classes, lectures, and hospital
attendance which are necessary for the ordinary
diplomas, but at each of the hospitals special
arrangements can be made for separate courses.
400 THE HOSPITAL. Sept. 3, 1904.
All that is here attempted is to give a more or less
general impression of the various medical schools ;
for further information and particulars application
should be made to the individual institutions by
letter addressed to " The Dean of the Medical School."
LONDON MEDICAL SCHOOLS.
All the teaching hospitals in London are now
schools in the faculty of medicine of the University
of London, and provide complete courses of study
for the preliminary scientific, intermediate, and final
M.B. examinations of the University, as well as for
the final examinations of the Universities of Oxford,
Cambridge, and Durham, and all the examinations
of the Conjoint Board of the Koyal Colleges of
Physicians and Surgeons, and the Society of Apothe-
caries.
In the case of a student about to enter at a
London medical school, the question of residence
may offer some difficulty. There are manifold dis-
advantages attaching to lodgings where a youth,
perhaps fresh from school, can live free from effec-
tive supervision. The ideal thing, if expense be not
a matter of great importance, is for him to board
with some member of the teaching staff, as there
usually are some of the younger members who are
willing to take in resident pupils on suitable recom-
mendations. If such a course be impossible, and
there are no friends or relatives with whom the
student can live, he may decide to take quarters in
the residential college, if there happen to be one
attached to the medical school of his choice, or fail-
ing this to take rooms. At most of the teaching
hospitals registers are kept of suitable lodgings, and
it is advisable that use should be made of these
before any particular rooms are decided upon.
St. Bartiiolomeav's Hospital Medical School.
St. Bartholomew's is the oldest and the second
largest hospital in London, and is, perhaps, the
leading medical school.
The site has been recently enlarged by the pur-
chase of acres of adjoining ground, and the
foundation stone of the first block of new buildings
was laid by the King on July 6. We understand
that the erection of this block will not interfere
with the work either of the hospital or the school.
The hospital contains 670 beds, in addition to 70
beds for convalescent patients at Swanley, in Kent,
and therefore there is an abundance of clinical
material. Several entrance scholarships are awarded
annually, and the total value of 'scholarships and
prizes offered every year amounts to upwards of
?790. There is a college for resident students
within the precincts of the hospital for admission to
which a recommendation from one of the medical
officers of the hospital is required.
All full and university students must become
members of the Students' Union on their entrance
to the medical school. The subscription is 10 guineas
for full students and six guineas for university
students. On payment of the above fee the student
becomes a life member of the Abernethian Society
and of the cricket, football, and other clubs.
The composition fee to the medical school for
students commencing their medical studies is pay-
able as^ follows :?30 guineas at commencement of
anatomical and physiological studies, and 30 guineas
annually for the five years of the medical curriculum.
On qualification at the end of five years a student 18
not liable for any further fees and receives a per-
petual ticket. Should he fail to qualify in this tinje
the fee for further instruction and attendance on the
practice of the hospital is 10 guineas for six months-
There are smaller fees for university students and
for those who desire to attend special courses ony
without becoming full students of the hospital.
Charing Cross Hospital Medical School.
Situated in the very centre of London, this
hospital has perhaps an advantage over all other
similar institutions in the metropolis as regards
accessibility. It is within two minutes' walk of tb0
Charing Cross terminus of the South Eastern Rail"
way and the Charing Cross Station of the Metro*
politan Railway, while omnibuses connect it with
every part of London. ,
Extensive additions are at present in course ot
construction, including new surgical wards, out"
patient department, operating theatres, and isolation
wards, which will increase the beds which the
hospital now possesses, including the convalescent
home at Limpsfield, to 300.
The medical school occupies a separate building 10
Chandos Street, immediately opposite to the hospital'
It was built in 1881 and enlarged 1889, and agai?
this year, owing to the increase in the number ot
students.
Several entrance scholarships are offered annually
for competition. Missionary studentships are als?
obtainable by clergymen through the Society for the
Promotion of Christian Knowledge.
There is a Students' Club, whose recreation ground
is at Eltham.
The composition fee?including subscription to the
students' club?is ?115.
St. George's Hospital Medical School.
After much deliberation and inquiry the Governors
have now decided to retain the hospital on ifcS
present site at Hyde Park Corner.
The hospital contains 350 beds, of which 205 are
devoted to surgical and 146 to medical cases, i?
addition to which there is a large convalescent hom0
of 100 beds at Wimbledon.
There are several entrance and other scholarships'
notably one of ?100 in Arts, open to candidates who
have not commenced their first winter attendance at
a metropolitan medical school, the subjects a111
scope of the examination being the same as for the
matriculation of the University of London ; another
of equal value in Science, the subjects being thos0
of the Preliminary Scientific (1st M.B.) examination
of the University of London, and one of ?50 f?r
students who have already passed a qualifying
examination in Anatomy and Physiology.
There are numerous appointments open to tb0
senior students, and amongst others may be special/
mentioned the following paid appointments, which ?re
open to students after they have held house office
Two Medical Registrarships ... salary ?200 per anno1*1
Surgical Registrarship   200
Curatorship of the Museum  ? 200
Assistant Curatorship   ,, 100
Resident Obstetric Assistantship ... ? 50
Two Demonstratorships of Anatomy ,, 50
Senior Anaesthetist ... ... ... ? 50
Two Junior Ano33thetists   ? 30
The composition fee is ?150.
1
Sept. 3, 1904. THE HOSPITAL. 401
Guy's Hospital Medical School.
The medical school of Guy's Hospital dates
irom 1768, at which time it was agreed to open
'he practice of the hospital to the pupils attached
the neighbouring St. Thomas's Hospital. This
association continued till 1825, during which period
*he two institutions enjoyed the title of the United
borough Hospitals. After this date the two
Medical schools become distinct.
Guy's Hospital contains 602 beds, and a note-
worthy feature is the reservation of certain wards,
Containing 40 beds in all, for cases of special clinical
Merest.
There is a college which accommodates about 60
indents, who are under the supervision of a resident
harden. The rooms are furnished, and the cost of
residence is moderate.
There is a remarkably well-conducted students'
c^b, which, perhaps, accounts in great part for the
??od fellowship that is so observable among Guy's
^en. By paying an annual subscription of two
tineas any student of the hospital obtains the
Privileges of membership of all the clubs, including
he students' club.
T Situated on the south side of the river near
London Bridge, the hospital ministers to a very
!arge population ; there is, therefore, plenty of work
0 be done, and the house appointments are especially
Suable.
The composition fees are 16 guineas for the first
^elve months or less period, and subsequently
V guineas a year till the student is qualified.
King's College Hospital.
The medical is only one of the many faculties for
which educational arrangements are provided at
Ring's College ; as a medical school King's College
hospital ranks very high, the instruction in the
Various branches of science, especially in anatomy
physiology, being probably unsurpassed in
London.
, The hospital, which contains 220 beds, is situated
^ Portugal Street, Lincoln's Inn, and is about five
l^&utes' walk from the college. In connection with
{he hospital is a convalescent home, containing 40
eds, at Hemel Hempstead.
. ^V"e mentioned last year that it was probable the
^ospital would be moved to the south of London in
he near future. "We are glad to be able to announce
hat an ample site has been secured in the neigh-
bourhood of Denmark Hill, and the new hospital
Which will be built there will be a spacious one, with
6very modern requirement for both patients and
indents. The teaching of the preliminary subjects,
^eluding chemistry, physics, biology, anatomy, and
Physiology, will continue to be given at King's
^?Uege in the Strand, and students entering now
Will by the time they are sufficiently advanced to
?tudy in the wards be able to do so in the new
b?spital.
Several entrance scholarships are offered for com-
petition every year, including two of the value of
-5 per annum, each tenable for four years, and one
\r *100. There are several others of smaller value.
Medical missionary studentships are also obtainable
hrough the Society for Promoting Christian Know-
^80.
The composition fee is ?135. Full particulars and
prospectus can be obtained by applying to Professor
Halliburton, Dean of the Medical Faculty.
The London Hospital Medical School.
Of all the large general hospitals this is perhaps
the one that is brought most prominently before the
notice of the general public. It is the largest insti-
tution of ita kind, and being situated in the East
End of London, it ministers to a very large and poor
district.
Several entrance scholarships are offered annually,,
including the Price scholarship in science, ,?120 ; the
Epsom Scholarship, ?126 ; and entrance scholarships
in science, anatomy and physiology, and arts. The
number of appointments open to qualified students is
large, and some of them are of considerable value
owing to the opportunities which they provide for
attaining a wide experience.
There is a Clubs' Union, embracing the athletic
and all the other clubs, to which all students are
expected by the College Board to belong. The
recreation ground is at Higham3 Park, and special
arrangements have been made with the Great
Eastern Railway for tickets at reduced rates for
members of the union.
The composition fee is 120 guineas if paid in one
sum, or 130 guineas if paid in three annual instal-
ments. A reduction of 15 guineas is allowed in the
case of sons of medical men.
St. Mary's Hospital Medical School.
Situated near Paddington Station, St. Mary's
Hospital is in a most convenient position for
those students who desire to live in the West End -r
moreover the hospital is in a very accessible position
owing to the proximity of the Great Western, Great
Central, Metropolitan, District, and Central London
Railways.
The hospital at present contains 281 beds, which
number will be raised to 350 on the opening of the
Clarence Wing, which will shortly be ready for
occupation. This extension will also provide new
operating theatres, a medical clinical theatre, an
obstetric department for cases of difficult labour, and
a complete electrical and ce-ray department.
Six entrance scholarships in natural science are
offered annually, and include one of ?145 open to
any student who has not completed a Winter Session
of medical study at a medical school, two of
60. guineas each open to students of any British
University, and one of ?145 open to students from
Epsom College, being the sons of medical men, who
have not completed a Winter Session of study at a
medical school. This scholarship is not awarded by
examination.
The composition fee is ?140 if paid in one sum on
entering the Medical School, and ?145 if paid in
four instalments. On payment of the full composi-
tion fee, and provided that he obtains a registrable
medical qualification within seven years from the
date of registration, the student becomes a perpetual
pupil, and is entitled to unlimited attendance on the
practice of the hospital. The composition fee for
students who have passed their examination in ana-
tomy and physiology is 60 guineas if paid at entrance,
65 guineas if paid in two annual instalments.
The Middlesex Hospital Medical School.
The hospital, which is in Mortimer Street, W.,
contains 340 beds, including special wards for children
402 THE HOSPITAL. Sept. 3, 1904.
and the diseases of women. There is a cancer wing
-which has 40 beds, and special research laboratories,
and the hospital therefore offers unequalled oppor-
tunities for the study of this disease, both clinically
and pathologically.
A complete electrical and ?c-ray department has
recently been provided in a temporary building which
has been erected in the courtyard of the hospital.
The hospital has the advantage of possessing a
residential college which faces on the hospital garden.
It is capable of accommodating about 30 students.
There is an amalgamated students' club, which all
general and dental students are required to join on
their entrance to the medical school. The subscrip-
tion is two guineas for general and one guinea for
dental students.
Several entrance and other scholarships and prizes
are offered annually.
The composition fee, if paid in one sum on entrance,
is 135 guineas. It may be paid, if preferred, in three
instalments, viz. : On entrance, 60 guineas ; at com-
mencement of second year, 50 guineas ; at commence-
ment of third year, 35 guineas.
St. Thomas's Hospital Medical School.
St. Thomas's Hospital contains 602 beds, of which
about 540 are in constant use.
There are numerous scholarships and prize3 in
connection with the school.
The various students' clubs were amalgamated in
1888. The annual subscription for membership of
the amalgamated clubs is ?2 10s. for the first three
years and 2 guineas for the years following. After
five consecutive subscriptions the student becomes a
life-member, provided he has obtained a registrable
qualification.
The composition fee covering the work of the first
year is 20 guineas. Students who have passed the
preliminary scientific or a corresponding examination
pay an entrance fee of 20 guineas and an annual
fee of 30 guineas, which is due in advance on the
first day of the term in which the student enters
and on the corresponding day of each successive year,
until a registrable qualification has been obtained.
A student who qualifies within six months of the
?date on which his last annual composition fee became
due is allowed a rebate of 15 guineas.
The entrance fee to students who have passed the
intermediate M.B.Lond. or a corresponding examina-
tion is 10 guineas. The annual composition fee
until qualification is 30 guineas.
University College, London (Faculty of
Medicine), and University College Hospital.
University College, like King's College, is com-
pletely equipped for providing educational facilities
in a wide range of subjects, of which medicine is
?one. The medical school is very flourishing, and
takes a high place among its fellow institutions in
London.
The hospital is, through the generosity of Sir J.
Blundell Maple, Bart., being rebuilt, and it is
thought that the whole of the new hospital will be
in working order within 12 months. There will
be accommodation for nearly 300 in-patients, with
extensive and well-equipped out-patient and special
-departments.
Scholarships and exhibitions of the value of ?800
are offered for competition every year, including the
Backnill Scholarship of ?120 and two entrant0
exhibitions of the value of 60 guineas each (examin*'
tion begins on September 20th, 1904, at 10 o'clock))
the subjects for which are chemistry, physics, botany>
and zoology, two exhibitions of 80 guineas each i?
anatomy and physiology, the Atchison Scholarship 0
?110 (awarded for general proficiency), and many
others.
Composition Fees :?For the courses required by
the University of London, 165 guineas, payable &
follows : For the preliminary scientific course, ^
guineas; for the intermediate, 60 guineas, or in
instalments of 37 and 25 guineas ; and for the fi?a
M.B. course, 80 guineas, or in two instalments of
and 32 guineas.
For the medical education required by
Examining Board in England and the Society 0
Apothecaries :?For the course required for the fir9
examination, 30 guineas, entitling to one attendance'
For the course required for the second examination
50 guineas (if paid in one sum), 51 guineas (if Pftl
in two instalments), as follows : First year, 31 guinea-^
second year 20 guineas (this fee entitles to attend'
ance during three years). For the course require0
for the third examination, 80 guineas (if paid in oDe
sum), 82 guineas (if paid in two instalments), ^
follows : First year, 50 guineas ; second year, ^
guineas.
Westminster Hospital Medical School.
The Westminster Hospital stands opposite
Westminster Abbey. .
It was founded in 1715, and first opened at a sma^
house in Birdcage Walk, under the name of tb?
"Publick Infirmary for the Sick and Needy." ^
the time of it3 commencement the only hospitals f?r
the relief of the sick poor in London were St. B*r'
tholomew's and St. Thomas's.
Westminster Hospital is notable in that it
the first institution of its kind in London which
founded by the voluntary contributions of the pubUc'
After occupying several different sites it was est?'
blished where it at present remains in 1834.
There is accommodation for upwards of 200 i?'
patient^.
There is a students' club union, the subscript011
for membership of which is included in the gener*
fees.
The composition fee is 110 guineas.
London (Royal Free Hospital) School of
Medicine for Women.
The Royal Free Hospital is in Gray's Inn Ro?^'
and the London School of Medicine for Women is lfl
Hunter Street, Brunswick Square, W.C. , .
There are 170 beds in the hospital, all of whic
are available for clinical teaching.
There are separate departments for the variou
branches of medical work, and arrangements h**
been made for students to attend the practice of
number of special hospitals, including the f0^
hospitals of the Metropolitan Asylums Board
the hospitals under the Asylum3 Board of ^
London County Council. i
There are numerous scholarships and prizes off0retf
annually in connection with the school, anaoQv
which may be mentioned the School Scholarship ??
?30 and the Sfc. Dunstan's Medical Exhibition 0
?60 a year for three years, extendable to five yea*"3'
Sept. 3, 1904. THE HOSPITAL. 403
Assistance is offered by several societies to ladies
^ho intend to work subsequently as medical
missionaries
The composition fee for the entire course after the
Preliminary Scientific Examination for the M.B. of
the London University and the Royal University
?f Ireland is ?135, and that for the Preliminary
Scientific Course is 33 guineas.
The composition fee for the course of the M.B.
Durham, for the diploma of the conjoint colleges of
Scotland, or the Society of Apothecaries, including
elementary science, is ?140.
PROVINCIAL MEDICAL SCHOOLS.
As we have stated above there are some advantages
^hich provincial schools possess over those of the
metropolis, and among them may be mentioned the
greater facility with which a degree can be obtained
from those provincial schools which are connected
^ith local universities. Of course the kudos attached
to the London degrees is high, but it should be
remembered that the examinations are very severe,
and failure to pass on one or two occasions may so
prolong the course as to entail considerable pecuniary
hardships and disappointment.
The provincial medical schools at which a com-
plete medical education can be obtained are those of
Birmingham, Bristol, Cambridge, Newcastle (Uni-
versity of Durham), Leeds, Liverpool, Manchester,
and Sheffield. Certificates of attendance at any of
these schools are accepted by the Royal Colleges,
and also by the University of London.
The University of Birmingham (Faculty of
Medicine).
To obtain a medical degree in the University of
Birmingham it is necessary as a rule to spend the
first four years of the five years' curriculum in the
tlniversity, but the Senate has power of recognising
attendance at another university as part of the
attendance qualifying for these degrees, and of
recognising examinations passed at such other
University as exempting from the examinations in
chemistry, physics, and comparative anatomy. In
the case of such students at least three years must
spent in attendance upon classes at the University.
The fifth year may be spent at any other school or
schools of medicine recognised by the University.
In order to obtain the degrees of M.B. and Ch.B.
five examinations have to be passed. The first is in
chemistry and physics; the second in anatomy,
comparative anatomy, and physiology; the third in
pathology and bacteriology; the fourth in forensic
Medicine, toxicology, and public health; and the
fifth, or final, in medicine, surgery, midwifery,
gynaecology, therapeutics, mental diseases, and
ophthalmology. The University grants degrees also
dental surgery and public health.
The General Hospital and the Queen's Hospital,
containing between them upwards of 400 beds, are
amalgamated for the purpose of clinical instruction,
^hich is carried on under the direction of the
Birmingham Clinical Board.
The composition fee for the whole course of
|ectures and instruction at the University is ?85,
besides which there is a composition fee for attend-
ance on the medical and surgical practice and the
clinical lectures at both hospitals of ?42, making a
total of ?127.
University College (Faculty of Medicine),
Bristol.
Students of the college are admitted to the clinical
practice of the Bristol Royal Infirmary and the
Bristol General Hospital conjointly. The infirmary
and the hospital comprise between them a total of
470 beds, and both are provided with extensive
out-patient departments and special departments.
The practice of the Bristol Royal Hospital for Sick
Children and Women, which contains 104 beds, and
that of the Bristol Eye Hospital, with 40 beds, are
also available.
The total number of beds open for clinical
instruction is therefore 614.
The composition fee for the lectures is 70 guineas,
and that for hospital practice is 35 guineas.
Special fees are charged for clerkships and dresser-
ships, and the total fees payable, including these,
amount to 132 guineas.
University of Cambridge.
In order to obtain the degree of Bachelor of
Medicine a student must become a matriculated
student of the University, reside in the University
the required portion of each of nine terms, pass or
obtain exemption from the previous examination,
pursue medical study for five years, and pass three
examinations.
Instruction in clinical medicine, surgery, and mid-
wifery is given at Addenbrooke's Hospital, so that it
is possible to obtain a complete medical education at
Cambridge ; but the usual practice is for men, after
spending three or four years at the University, and
passing their examinations in anatomy and physi-
ology, and in pharmacology and general pathology,
to go to London and finish their clinical work there ;
and this is no doubt the best course to pursue.
University College, Cardiff.
Since it was founded, in 1883, University College,
Cardiff, has prepared students for the Preliminary
Scientific examination of the University of London,
and for the corresponding examinations of other
licensing bodies. In 1893, Chairs of Anatomy and
Physiology and a Lectureship in Materia Medica
and Pharmacy were established, making it possible
to spend three out of the five years of the medical
curriculum at this school.
Arrangements have been made with the managing
committee of the Cardiff Infirmary to give students
of the college the privilege of attending this hospital,
which contains 180 beds.
The composition fee for the three years' courae of
study for the Preliminary Scientific and Intermediate
Examination for the London M.B. is ?57 10s.
The University of Leeds (School of Medicine).
Clinical instruction is given in the general
infirmary, which contains 480 beds, and is amply
provided with special departments.
Students of the University also have access for
instruction to the Leeds Public Dispensary, the City
Fever Hospitals, the Hospital for Women and
Children, and the West Riding Asylum at Wake-
field.
The School of Medicine, which is particularly well
404 THE HOSPITAL. Sept. 3, 190*?
equipped in every way, is separated from the in-
firmary by a street only, an arrangement which
facilitates attendance at both institutions.
The composition fee for university students who
have already taken the course of instruction for the
first M B. examination, and for non-university
students who have taken out the required instruc-
tion in chemistry, chemical physics, and elementary
biology, is 64 guineas, and that for the hospital
practice is 40 guineas.
University College (Medical Faculty),
Liverpool.
Students are prepared for the degrees of the Uni-
versity of Liverpool, or may study for the degrees
and qualifications of various other licensing bodies.
The laboratories are very well equipped, and the
facilities for instruction are most complete.
The clinical work may be done at the Royal
Infirmary, which contains 300 beds, or at the United
Hospitals Clinical School, consisting of the David
Lewis Northern Hospital, where there are 200 beds,
the Royal Southern Hospital containing 200 beds, or
the Stanley Hospital with 104 beds.
The lying-in, eye and ear, children's, and other
special hospitals are also .open to students, and
courses of instruction in tropical diseases can be
obtained at the Liverpool School of Tropical
Medicine. Fellowships, scholarships, and prizes of
over ?700 are awarded annually.
Tiie "Victoria University of Manchester
(Medical Faculty).
The medical department of the University is par-
ticularly well provided with facilities for teaching the
preliminary subjects?namely, anatomy, physiology,
pathology, and materia medica.
Clinical instruction is provided at the Manchester
Royal Infirmary, which contains 290 beds, and which
has large out-patient and casualty departments.
Associated with the Infirmary is a Convalescent
Hospital at Cheadle containing 136 beds. Women
students are admitted to the practice of the Infir-
mary on the same terms as men.
Three halls are licensed by the college for the
residence of students?namely, Dalton Hall, Victoria
Park, and Hulme Hall, Plymouth Grove, for men,
and Ashburne House, Victoria Park, for women.
The composition fee for the full course of medical
study required for the ordinary qualifications is ?70,
in addition to which the fee for hospital practice is
?42.
University of Durham (College of Medicine,
Newcastle-upon-Tyne).
For students who intend to graduate at the
Durham University it is necessary that one of the
five years of professional education should be spent
in attendance at the College of Medicine, Newcastle-
upon-Tyne. During the year so spent the student
must attend the medical and surgical practice and
clinical lectures at the Infirmary, which contains 280
beds. No residence at Durham is necessary. The
remaining four years of the curriculum may be spent
at Newcastle-upon Tyne or at one or more of the
recognised medical schools.
The composition fee for the complete course of
lectures is 72 guineas, and for the medical and
surgical practice of the hospital 25 guineas.
Oxford University.
One of the chief events in the recent history o
Oxford University has been the revival of the
Medical School. Admirable arrangements now exist
for instruction in the preliminary medical sciences ,
while a certain proportion of the clinical work can
be done, if desired, in the wards of the Radclifle
Infirmary, previous to taking out the full course in
the metropolis. In order to obtain the Oxford
medical degree it is necessary to be a graduate id
Arts of the University.
University College Sheffield, (Department of
Medicine).
The Medical Department is close to the Arts and
Science Department of the university, and is within
easy distance of the Royal Infirmary, the Roya*
Hospital, the Jessop Hospital for Women, and the
City Fever Hospital. It possesses well-equipped
laboratories and other necessaries for the preliminary
studies, and a large field for clinical work in the
above-named hospitals. The courses of instruction
are recognised by the various examining bodies.
The composition fee is 60 guineas if paid at the
commencement of medical study ; but it may be pa1''
in two instalments of 35 guineas at entrance, and
30 guineas at the beginning of the second year ot
study. These fees do not cover medical and surgic&j
hospital practice and clinical work. An additional
fee of ?45 will entitle a student to a perpetual ticket
for hospital practice, excepting that of the Jessop
Hospital, the Fever Hospital, and the South York-
shire Asylum, the fees for which must be paid
separately.
SCOTTISH MEDICAL SCHOOLS.
University of Edinburgh.
To obtain the Edinburgh medical degree it 15
necessary to pass the Preliminary Examination or
it3 recognised equivalent, and to pass four profes*
sional examinations. The first is in botany, zoology*
physics, and chemistry ; the second in anatomy*
physiology, materia medica, and therapeutics ; the
third in pathology ; and the final in surgery, clinica'
surgery, medicine, clinical medicine, midwifery>
medical jurisprudence, and public health.
The degree of M.B. is not conferred separately
from the Ch.B. In order to obtain the degree it i?
necessary to spend at least two of the five years oi
medical study in the University of Edinburgh.
Clinical instruction is given in the Royal Infirmary)
which contains nearly 900 beds ; Royal Hospital f?r
Sick Children, with 120 beds; Maternity Hospital;
City Fever Hospital; and the Royal Morningside
Asylum.
School of Medicine of the Royal Colleges,
Edinburgh.
This School of Medicine is composed of lecturer?
licensed by the Royal Colleges of Surgeons and
Physicians, and also recognised by the University)
who lecture in several buildings near the Roya*
Infirmary.
The teaching is similar to that of the Scottish
Universities, and the students receive similar certifi'
cates at the close of each session.
The facilities for clinical instruction are similar to
those provided for the University.
Sept. 3, 1904. THE HOSPITAL. 405
Medical College foe Women, Edinburgh.
Precisely the same facilities for medical study are
given as to male students in the School of Medicine,
Edinburgh. Regulations, fees, and arrangements
are the same. The lecturers are recognised by the
University of Edinburgh as giving courses admitting
to the degrees of M.B., Ch.B. Clinical instruction
is given in the Royal Infirmary and other hospitals
mentioned above.
University of Glasgow.
The whole course of study for the M.B , Ch.B., can
be passed in the Medical School of the University.
The regulations are similar to those given above
for Edinburgh. Clinical instruction is given at the
Western Infirmary, which contains 420 beds, and
also at the Lunatic Asylum, the Eye Infirmary, the
Maternity Hospital, the City Fever Hospitals, and
various other special hospitals.
Queen Margaret College, Glasgow
(the Women's Department of the University).
This is an integral part of the University of Glas-
gow. The courses, regulations, and fees are the same
as for men. The instruction is given by University
Professors and Lecturers appointed by the Uni-
versity Court. The college provides a separate
Wlding, with class-rooms, laboratories, library, and
^ther teaching appliances. The women have all the
flghts and privileges of University students, and do
^eir clinical work in the Royal Infirmary, where
special wards are reserved for their use, and in other
sPecial hospitals.
St. Mungo's College, Glasgow.
The college buildings, which are provided with
^ass-room and laboratory accommodation for three
0r four hundred students, are situated within the
founds of the Royal Infirmary, the wards of which
available for clinical instruction, as also are those
the Western Infirmary. In connection with the
former there are pathological and bacteriological
laboratories, and a large pathological museum. All
^e classes in this college qualify for the diplomas of
English, Scottish, and Irish Conjoint Boards.
Anderson's College Medical School,
The college, at which a full medical curriculum
c&n be obtained, is situated in Dumbarton Road,
close to the Western Infirmary and within four
Minutes' walk of the University.
Degrees and diplomas : Certificates of attendance
^ the lectures are accepted by the Universities of
London and Durham, by the Royal University of
Iceland, and by all the Royal Colleges and licensing
boards in the United Kingdom.
University of Aberdeen.
_ The regulations are practically the same as those
?iven for Edinburgh. Clinical instruction is obtained
lt* the Royal Infirmary, which contains 250 beds, in
the Sick Children's Hospital, and several other insti-
tutions, including the Royal Lunatic Asylum and the
^ity Fever Hospital.
University of St. Andrews (University College,
Dundee, and United College, St. Andrews).
In order to graduate, at least two of the five
years of medical study must be spent either at the
United College, St. Andrews, or at University
College, Dundee. The remaining three years may
be passed at other medical schools, but the whole
course may be completed, if preferred, at University
College, Dundee.
The new medical buildings in University College,
Dundee, will be opened in October.
IRISH MEDICAL SCHOOLS.
The School of Physic in Ireland,- Dublin.
This school is formed by an amalgamation of the
medical school of Trinity College and of the Royal
College of Physicians.
Clinical instruction is given by the staff of Sir
Patrick Dun's Hospital, and courses of clinical study
can be carried out at many of the other hospitals in
Dublin.
The Catholic University Medical School.
Complete courses of lectures, as required by the
examining bodies, are provided.
For all further information about the school
application should be made to the Registrar, Medical
School, Cecilia Street, Dublin.
Royal College of Surgeons in Ireland (The
Schools of Surgery).
These schools are attached by charter to the
Royal College of Surgeons. They are carried on
within the college buildings, and are specially
subject to the supervision and control of the Council,
who are empowered to appoint and remove the
professors, and to regulate the methods of teaching
pursued. The buildings have been reconstructed,
the capacity of the dissecting-room nearly trebled,
and special pathological, bacteriological, public health,
chemical, and pharmaceutical laboratories fitted with
the most approved appliances, in order that students
may have the advantage of the most modern methods
of instruction. There are special rooms set apart
for lady students.
All the lectures and courses of practical instruc-
tion may be attended by medical students who are
otherwise unconnected with the college.
Full information is to be had on application to the
Registrar, Royal College of Surgeons, Dublin.
Queen's College, Belfast.
The courses of study meet the requirements of the
Royal University of Ireland and other examining
bodies.
Clinical instruction is given at the Royal Victoria
Hospital and other hospitals.
Full information can be had on application to the
Registrar, Queen's College, Belfast.
Queen's College, Cork.
Students can attend courses which meet the
requirements of the Royal University of Ireland,
the Conjoint Boards of London, Edinburgh, or
Dublin, and other examining bodies.
Clinical instruction is given at the North and
South Infirmaries, each of which contains 100 beds,
and at other hospitals.
Further information can be had on application to
the Registrar, Queen's College, Cork.
406 THE HOSPITAL. Sept. 3, 1904.
MEDICAL SCHOOLS AND MEDICAL
QUALIFICATIONS FOR WOMEN.
The following are the qualifying bodies which
admit women as candidates for thsir degrees or
diplomas :?all the Universities of England with
the exception cf the Universities of Oxford and
Cambridge; the Universities of Scotland ; the Royal
Universities of Ireland ; the Society of Apothecaries,
London; and the Conjoint Board of the Royal Col-
leges of Physicians and Surgeons of Scotland and
Ireland.
The regulations of the General Medical Council in
regard to medical edusation and examinations apply
to women exactly as they do to men.
With regard to place of study, women medical
students can obtain a complete medical education by
becoming students of the London School of Medicine
for Women, which is connected with the Royal Free
Hospital; the Medical College for Women, Edin-
burgh ; or Queen Margaret College, Glasgow (which
is now an integral part of the University of Glas-
gow), all of which are open to women only ; or they
may study at the Schools of Surgery of the
Royal College of Surgeons in Ireland, where
special rooms are set apart for lady students ; or at
one of the Queen's Colleges at Belfast, Cork, and
Galway. They can also enter at the Universities of
Edinburgh, Aberdeen, and St. Andrews; at the
Universities of Birmingham, Leeds, and Manchester,
and at the University of Durham College of Medi-
cine which is situated in Newcastle-upon-Tyne. The
medical classes of the University of St. Andrews are
open to women on an equal footing with men, and it
may be arranged either to take the first two years
at United College, St. Andrews, and the rest at
Dundee, or the whole five years may be spent at
University College, Dundee. The Dundee Royal
Infirmary offers facilities for clinical instruction to
women students.
For English women students who feel equal to the
examinations of the London University, the London
School of Medicine for Women will be found
thoroughly suitable, or,, they may spend a portion of
their time at Newcastle after matriculating at the
Durham University and then finish their course in
London. The Secretary of the London School of
Medicine for Women, 8 Hunter Street, London,
W.C., will give every information.

				

